# P-871. Outcome of Ceftriaxone resistant, *Escherichia-coli* and *Klebsiella spp*. bacteremia comparing Carbapenem and Beta-lactam/Beta-lactamase inhibitors in a public sector hospital from Pakistan

**DOI:** 10.1093/ofid/ofae631.1062

**Published:** 2025-01-29

**Authors:** Rohama Samar, Beena Rani, Zaheer Udin Babar, Asma Nasim, Sunil Dodani

**Affiliations:** Sindh Institute of Urology and Transplantation, Karachi, Sindh, Pakistan; Sindh Institute of Urology and Transplantation, Karachi, Sindh, Pakistan; Sindh Institute of Urology and Transplantation, Karachi, Sindh, Pakistan; Sindh Institute of Urology and Transplantation, Karachi, Sindh, Pakistan; Sindh Institute of Urology and Transplantation, Karachi, Sindh, Pakistan

## Abstract

**Background:**

Carbapenem are recommended for the treatment of Ceftriaxone (CRO) resistant Enterobacterales, however, there are concerns of cost and resistance. Our aim is to compare the outcome of CRO resistant E-coli and Klebsiella bacteremia between Carbapenem and Beta-lactam/beta-lactamase inhibitors (BL/BLI).

**Figure:**
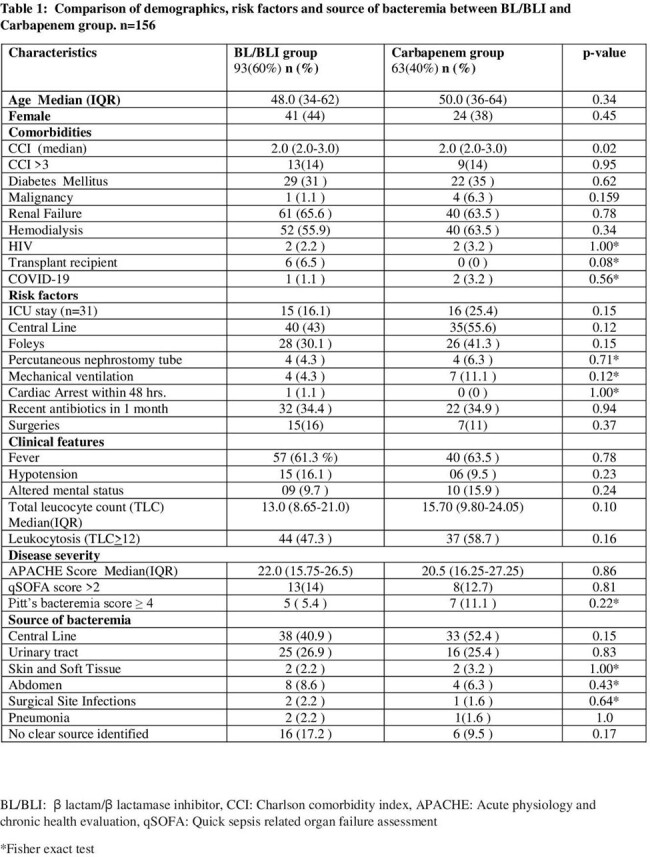

**Methods:**

A prospective cohort study conducted from October 2021 to June 2022. All adult patients with E coli or Klebsiella spp. bacteremia, CRO resistant and sensitive to both BL/BLI and Carbapenem were included. The patients were divided into BL/BLI and Carbapenem groups. Demographics, clinical features, comorbidities, laboratory parameters and intensive care unit stay were compared. Outcomes were bacteriological clearance, clinical success and all-cause mortality at day 14 of bacteremia.

**Figure:**
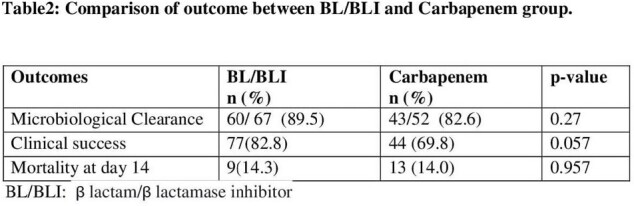

**Results:**

A total of 156 patients, 93(59.6%) in BL/BLI and 63(40%) in Carbapenem group were included. There was no difference in co-morbidities, risk factors and severity of disease. The 14 day all-cause mortality was 14.1%. No statistically significant difference was found between BL/BLI and Carbapenem group regarding bacteriological clearance (p=0.27) and mortality (p=0.95). The Carbapenem group had less clinical success rate (69.8% vs 82.8%, p=0.057),however not statistically significant.

**Figure:**
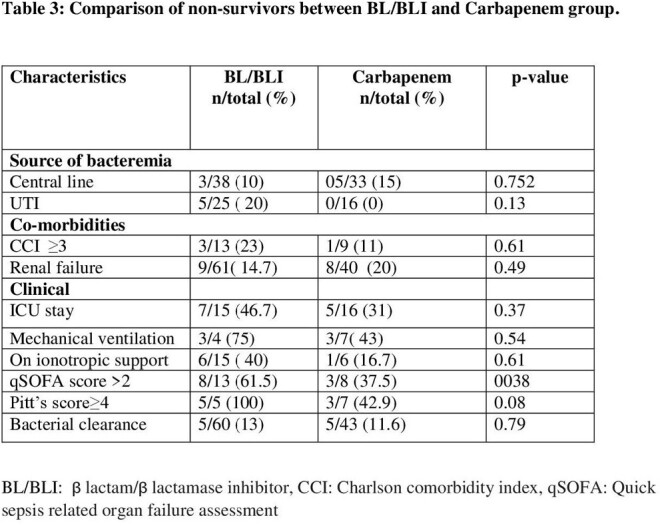

**Conclusion:**

BL/BLIs were as effective as Carbapenem in microbiological clearance, clinical success and mortality in CRO resistant E-coli and Klebsiella bacteremia.

**Disclosures:**

**All Authors**: No reported disclosures

